# Interactions between human immunodeficiency virus and human endogenous retroviruses

**DOI:** 10.1128/jvi.02319-24

**Published:** 2025-02-07

**Authors:** Mengying Li, Fengting Yu, Baoli Zhu, Jiang Xiao, Chang Yan, Xiaojie Yang, Xuelei Liang, Fang Wang, Hanxi Zhang, Fujie Zhang

**Affiliations:** 1 Medical School, University of the Chinese Academy of Sciences 74519 , Beijing, China; 2 Beijing Ditan Hospital, Capital Medical University 12638 , Beijing, China; 3 Clinical Center for HIV/AIDS, Capital Medical University 12517 , Beijing, China; 4 CAS Key Laboratory of Pathogenic Microbiology and Immunology, Institute of Microbiology, Chinese Academy of Sciences 85387 , Beijing, China; 5 WHO Collaborating Centre for Comprehensive Management of HIV Treatment and Care, Beijing Ditan Hospital, Capital Medical University 12638 , Beijing, China; Department of Microbiology, New York University, New York, New York, USA

**Keywords:** human immunodeficiency virus (HIV), human endogenous retroviruses (HERVs), poor immune reconstitution (PIR), HIV reservoir

## Abstract

Human immunodeficiency virus (HIV), a retrovirus of the Lentivirus genus, targets CD4^+^ T cells, causing immune dysfunction and AIDS. Approximately 8% of the human genome consists of human endogenous retroviruses (HERVs), ancient retroviral remnants that may interact with HIV. Despite antiretroviral therapy, challenges such as drug resistance, poor immune reconstitution (PIR), and reservoirs remain. This GEM discusses the impact of HIV on HERVs, the potential roles of HERVs in PIR and reservoirs, and provides insights into future research directions.

## INTRODUCTION

Human immunodeficiency virus (HIV), the most impactful exogenous retrovirus affecting the human population, compromises immune function, leaving individuals vulnerable to opportunistic infections and ultimately mortality (1). As a retrovirus, HIV undergoes genomic integration following infection, forming viral reservoirs and producing substantial quantities of virus upon discontinuation of antiretroviral therapy (ART) (2). Despite ART’s success in suppressing viral replication and restoring CD4^+^ T cell counts, HIV persists in resting memory CD4^+^ T cells, posing a major barrier to eradication (3). Poor immune reconstitution (PIR) affects 15–30% of virologically successful people living with HIV (PLWHs), resulting in persistently low CD4^+^ T cell counts and increased mortality (4, 5). In addition, the high frequency of recombination and mutation of HIV facilitates the emergence of drug-resistant strains, complicating diagnosis, and treatment efforts (6).

**Fig 1:**
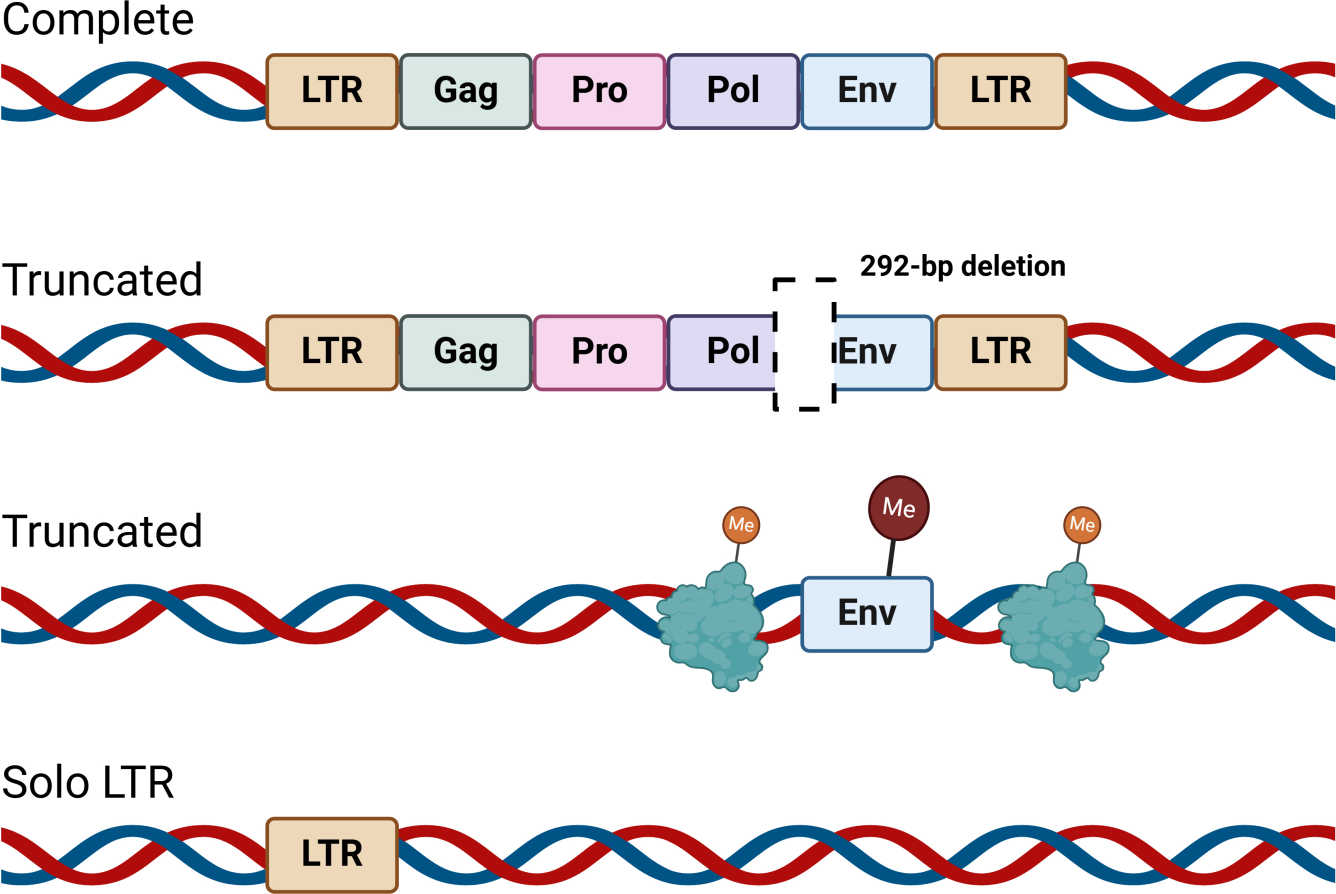
Integrated retroviruses. Complete HERVs are essentially identical to the integrated proviruses of exogenous retroviruses; they contain two LTRs and a full complement of coding sequences (*gag*, *pro*, *pol*, and *env*). Truncated refers to the deletion of specific sequences within HERV genomes due to mutations or recombination. For instance, the type 1 of HERV-K (HML-2) proviruses exhibit a 292 bp deletion at the *pol-env* junction, rendering them incapable of encoding Rec or Env proteins. Post-integration recombination events in HERVs may result in gene sequences retaining only certain coding regions, such as *env*. However, under normal physiological conditions, these sequences remain silenced through DNA methylation and/or histone methylation. Currently, the majority of HERVs exist as solitary LTRs. LTR, long terminal repeat; *Gag* encodes matrix, nucleocapsid, and capsid proteins; *Pro* encodes protease; *Pol* encodes reverse transcriptase and integrase; *Env* encodes envelope protein; and Me represents methylation. Created in https://BioRender.com.

Our ancestors have historically encountered various retroviruses, the remnants of which persist in the genome as transposable elements known as human endogenous retroviruses (HERVs), comprising approximately 8% of the human genome (7). HERVs proviruses share a gene structure similar to that of exogenous retroviruses, containing *gag*, *pro*, *pol*, and *env* genes, along with two long terminal repeats (LTRs) (8, 9). Over time, HERVs have become internalized within the genome, with recombination between LTRs leading to the excision of coding regions in many HERV family genes (8–10). Consequently, most HERV gene copies in the human genome exist as isolated LTRs (Fig. 1) (8–10). The *pol* region of HERVs bears significant similarity to the *pol* regions of exogenous retroviruses, such as *gamma-retrovirus*, *beta-retrovirus*, and *spumavirus*, enabling the classification of HERVs into three major groups (Table 1) (7). To date, more than 30 HERV families have been identified (11). Among these, the HERV-K (HML-2) family is the most recently integrated endogenous retrovirus in the human genome and is distinguished by its high level of gene sequence preservation and transcriptional activity (7). As such, HERV-K (HML-2) has become one of the most intensively studied HERV families. The identification of HERVs offers potential insights into an evolved defense mechanism that may protect humans from retroviral infections.

**TABLE 1:** The major family of human endogenous retroviruses

	Genus	Human endogenous retroviruses
Class I	*Gammaretrovirus*	HERV-W, HERV-H, HERV-E, HERV-R, HERV-F, HERV-I, HERV-P, HERV-T
Class II	*Betaretrovirus*	HERV-K
Class III	*Spumavirus*	HERV-L

In the ART era, key challenges such as recombination, drug resistance, PIR, and HIV reservoir hinder HIV treatment and research. HERVs may play a role in these processes. While studies indicate that HERVs respond to HIV infection, the precise nature of their interaction remains unclear. This GEM explores the impact of HIV on HERVs, the potential influence of HERVs in PIR and reservoirs, and highlights areas for future research.

## EFFECTS OF HIV INFECTION ON HERVs

### HIV infection alters the expression profile of HERVs

Previous studies have consistently shown that HIV infection upregulates the expression of HERVs. For example, the mRNA levels of HERV-K (HML-2) *gag* and *env* regions are elevated in peripheral blood mononuclear cells (PBMCs) of PLWHs in the late stages of HIV infection (12). Moreover, even in PLWHs with good immune status, the mRNA levels of HERV-K (HML-2) *env* region remain elevated in PBMCs, regardless of ART status (13). Another study reported that compared to PLWHs with successful ART, PLWHs with treatment failure had HERV-K (HML-2/3) RNA levels in plasma that were two orders of magnitude higher (14). A subsequent longitudinal study found that, in PLWHs with successful ART, HERV-K (HML-2) RNA titers were typically undetectable, although lower levels of HERV-K (HML-2) RNA could be detected in some individuals (15). In contrast, in PLWHs with treatment failure, the elevation of HERV-K (HML-2) RNA titers often preceded HIV rebound (15). Furthermore, a study examining the expression differences of HERV-K (HML-2) in HIV-1-infected individuals of different subtypes found that HERV-K (HML-2) *gag* region mRNA was significantly upregulated in subtype B-infected individuals, while *pol* region mRNA was significantly upregulated in CRF01_AE and CRF07_BC-infected individuals (16). However, no significant differences in the expression of the *env* region were observed between the groups (16). In contrast, a study found that HIV elite controllers exhibited significantly lower expression of HERV-K (ERVK-6) RNA in PBMCs compared to healthy controls and ART-treated PLWHs (17). The same study also showed that ART-treated PLWHs had reduced ERVK-6 RNA expression in PBMCs compared to healthy controls, though the difference was not statistically significant (17). These results suggest that the activation levels of different HERV-K subfamilies vary under different disease stages, immune statuses, and the influence of ART.

Additionally, one study found no significant difference in HERV-W *env* expression between PLWHs who received and not receive ART (18). Further *in vitro* drug treatment studies showed that, in monotherapy, only Efavirenz at the highest concentration (10 µM) reduced HERV-W Env expression, whereas in combination therapy (including Lamivudine, Tenofovir, Daranuvir, Efavirenz, and Raltegravir), a reduction in HERV-W Env expression occurred at a concentration as low as 1 µM (18). In contrast, another study demonstrated that, compared to healthy controls, the expression of HERV-H in the peripheral blood of ART-naive PLWHs was significantly decreased, while the expression of HERV-I, HERV-L, HERV-W, HERV-K (HML-1), HERV-K (HML-2), HERV-K (HML-3), HERV-K (HML-5), and HERV-K (HML-6) was significantly increased in the PBMCs of ART-treated PLWHs (19). This study further demonstrated, through *in vitro* cell experiments, that the expression changes of the HERV family were widespread across different cell lines following HIV infection, and these changes exhibited a clear time-dependent pattern (19). In addition, another study using pseudovirus stocks and cell line models showed that HIV increases the expression of HERV-K RNA in a dose-dependent manner, with consistent results across different pseudovirus stocks (20). In this study, the researchers also used different pseudovirus models to simulate chronic and acute HIV infection phases, revealing that HERV-K expression may be increased during both stages (20). Notably, since the U87MG cells used in the experiments inhibit HIV replication, it is suggested that HIV may promote the expression of HERVs through its viral components (20). Another related study demonstrated that the expression of HERV-T, HERV-E, HERV-W, ERV-9, and HERV-K (HML-3, HML-4, and HML-10) was upregulated in HIV-infected cells, and these genes displayed distinct expression patterns in cell models of persistent and acute infection (21). Nevertheless, other studies have shown that in HIV-infected cells, most HERVs are downregulated, with HERV-K (HML-2), which had previously shown significant upregulation, also exhibiting a decreasing trend (22).

Besides, studies have reported the detection of HERV proteins in PLWHs, which will be further discussed in subsequent sections. Although HERV virions have not yet been detected in PLWHs, HERV-K-like virions have been successfully isolated from patients with lymphoma, breast cancer, and melanoma (23, 24). Therefore, future studies may further investigate the presence of HERV virions in PLWHs. Taken together, these findings suggest that HIV infection may lead to dysregulated expression of HERVs, potentially occurring in a time-dependent manner.

### Mechanisms of HIV-induced alterations in HERVs expression

Subsequently, researchers have delved into the mechanisms underlying the dysregulation of HERV expression induced by HIV infection. Gonzalez-Hernandez et al. demonstrated, through luciferase reporter assays, that HIV-Tat regulates the expression of HERV-K (HML-2) gag by interacting with the promoter of the HERV-K (HML-2) *gag* gene, with transcription factors NF-κB and NF-AT involved in Tat-induced promoter activation (25). This team further treated PBMCs from healthy donors with Tat, extracted total RNA, and conducted transcriptome sequencing. The results revealed that Tat significantly activated the expression of 26 unique HERV-K (HML-2) proviruses, silenced 12 proviruses, and did not significantly alter the expression of other proviruses (26). Similarly, a study by Uleri et al. found that HIV-Tat interacts with extracellular TLR4, inducing TNF-α production, which indirectly stimulates the expression of MSRV and Syncytin-1, both members of the HERV-W family (27). Moreover, the response of MSRV and Syncytin-1 in PBMCs to Tat was found to be dependent on the cellular context. For example, in monocytes, Tat stimulates MSRV expression while inhibiting Syncytin-1 expression, whereas in differentiated macrophages, both are activated by Tat (27). Uleri et al. further discovered that epidermal growth factor (EGF) counteracts the Tat-induced regulation of HERV-W family members, suggesting that EGF may mitigate the neurotoxic effects of Tat-activated HERV-Ws, potentially providing neuroprotective effects (28). In addition, another research team conducted structural analysis and found that HIV-Rev shares similarities with HERV-Rec, indicating that HIV-Rev may promote HERV-K mRNA expression by mediating the nuclear export of HERV-RcRE (29, 30).

Besides, some researchers have suggested that opportunistic infections induced by HIV may be one of the mechanisms underlying the altered expression of HERVs (7, 31). Common opportunistic infections associated with AIDS include tuberculosis and nontuberculous mycobacterial infections, fungal infections (such as cryptococcal meningitis, pneumocystis pneumonia, and candidiasis), and viral infections (including cytomegalovirus, herpes simplex virus, and Epstein-Barr virus infections). Currently, there is a lack of studies investigating the changes in HERV expression in the context of tuberculosis, nontuberculous mycobacterial infections, and fungal infections. However, research has reported alterations in HERV expression in the setting of viral infections (31, 32).

Compared to healthy individuals, the immune system of PLWHs is often in a state of abnormal activation, with persistent inflammation being considered a major driver of non-AIDS-related events. Currently, factors such as viral reservoirs, depletion of regulatory T cells/dysfunction of immune suppression, and gut microbiota translocation/dysbiosis are recognized as key contributors to abnormal immune activation in PLWHs (33, 34). Prior studies have identified that certain ERV promoter regions contain interferon-stimulated response elements, while other promoters include conserved binding motifs for pro-inflammatory transcription factors, such as NF-κB (9). A murine *in vitro* experiment demonstrated that compared to wild-type mice and TLR3/9-deficient or double-deficient mice, TLR7-deficient mice exhibited dysregulated ERVs expression (35). In a mouse model of idiopathic autism, a positive correlation between the expression of ERVs, TLRs (such as TLR3 and TLR4), and pro-inflammatory cytokines (such as IL-1β, IL-6, and TNF-α) was observed (36). Moreover, bioinformatic analysis revealed that genes related to the JAK-STAT signaling pathway, including STAT1, STAT2, and IRF-1, are closely associated with HERV expression, suggesting that interferons may promote HERV elements’ expression (37). Similarly, studies have shown that under IFN-γ treatment, HERV-K (HML-2) expression is upregulated (38). Dopkins et al. found in their *in vitro* studies that activation of innate immune responses might induce HERV expression and further promote the generation of inflammatory cytokines such as TNF-α (39). Although the exact impact of inflammatory responses on HERV expression in HIV infection remains unclear, based on the aforementioned studies, it is hypothesized that the persistent inflammatory response triggered by HIV infection may be one of the potential mechanisms underlying HERV dysregulation. In conclusion, HIV may directly or indirectly regulate the expression of HERV.

## EFFECTS OF HERVs ON HIV INFECTION/PATHOGENESIS

Previous studies have shown that the expression products of HERVs can co-assemble with HIV to form defective viral particles. Co-expression of HERV-K Gag and HIV Gag alters the size and morphology of HIV particles, resulting in a decrease in HIV infectivity (40). Besides, based on ten full-length proviral sequences of HERV-K, researchers constructed a consensus sequence of HERV-K (termed HERV-KCON). The results indicated that HERV-KCON Gag could reduce the release efficiency of HIV Gag (41). Further investigation revealed that HERV-K108 Env also decreased the production of HIV viral particles (42). However, some studies have shown that HIV particles bearing HERV-W Env maintain infectivity and can infect CD4-negative cells (43, 44). These findings underscore the necessity for further research into the maturation of HIV particles and the identification of potential drug targets.

In addition to co-assembling with HIV, HERVs protein products may contribute to HIV replication, even in the presence of antiretroviral drugs. Researchers have observed both unprocessed and correctly processed HERV-K (HML-2) Gag and Env proteins in individuals with varying viral loads, suggesting active HERV-K (HML-2) protease (PR) activity (45). HERV-K (HERV-K10) PR may cleave HIV polyprotein substrates, potentially complementing HIV PR under drug resistance (46). Nevertheless, other studies show that HERV-K PR does not fully restore viral infectivity (47). Notably, the dUTPase required for DNA replication is predominantly localized in the nucleus and mitochondria, whereas the dUTPase encoded by HERV-K can be present in the cytoplasm (48). It has been hypothesized that the phenomenon may be related to the ability of RNA transcribed from integrated HIV DNA to be reverse transcribed into stable cDNA within the cytoplasm (48). We hypothesize that the activities of HERVs PR and dUTPase may provide supplemental functions to HIV replication, which could potentially play a role in the development of HIV drug resistance. However, this hypothesis remains to be validated through further studies, as the exact contribution of HERVs to HIV resistance mechanisms is yet to be fully understood.

**Fig 2:**
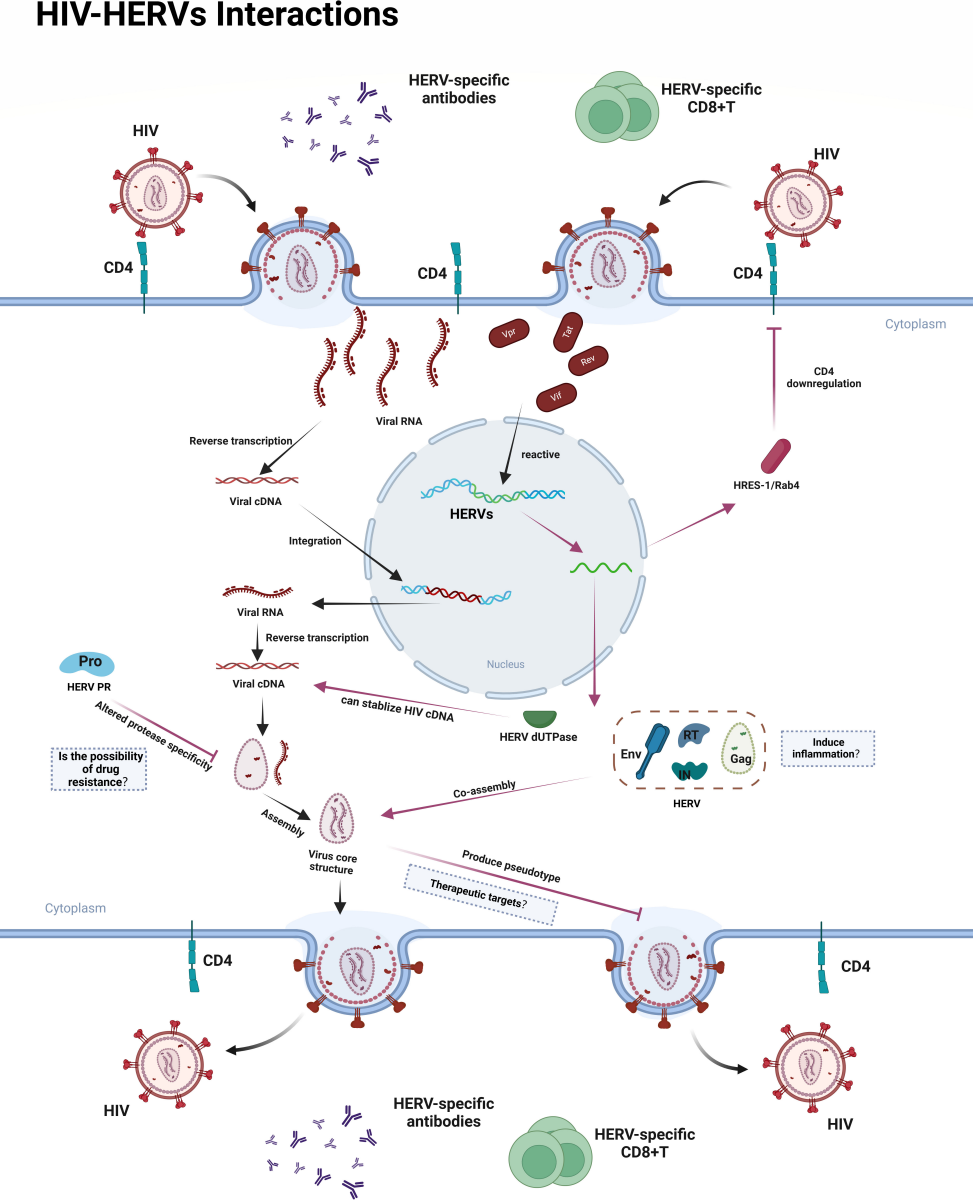
Interactions between HIV and HERVs. Research has shown that HIV proteins generated following reverse transcription and integration of the viral genome into the host genome, as well as the inflammatory state induced by HIV entry, can activate and transcribe HERVs. Currently, most HERVs exist as solitary long terminal repeats (LTRs), which possess regulatory functions and can upregulate the expression of certain antiviral genes. HERVs containing coding genes can produce retroviral proteins, many of which share high similarity with HIV proteins. These proteins may substitute HIV proteins and participate in HIV particle assembly, but in most cases, the resulting recombinant viral particles exhibit reduced infectivity. HRES-1/Rab4 (a protein encoded by HERVs) regulates CD4 expression by inhibiting its surface expression on peripheral blood CD4^+^ T cells and targeting it for lysosomal degradation. Additionally, the production of HERV proteins can stimulate the host immune system to produce anti-HERV antibodies and T cells, thereby contributing to the restriction of HIV replication. While HERVs exhibit beneficial antiviral functions for the host, there is evidence suggesting detrimental effects during HIV infection. For instance, HERV-K dUTPase may be able to stabilize reverse-transcribed HIV cDNA after integration. Black arrows: the process of HIV replication and its regulatory effects on HERVs. Red arrows: the influence of HERVs on HIV replication. Dashed box: hypotheses proposed in this study. Created in https://BioRender.com.

A novel transcript encoded by the antisense strand of HRES-1 (a member of the HERV family) was isolated from PBMCs, and the encoded protein was named HRES-1/Rab4 (49). Studies have shown that HIV Tat protein or HIV infection can increase the expression of HRES-1/Rab4, and overexpression of HRES-1/Rab4 inhibits HIV infection, gag p24 production, and apoptosis (49). Further research demonstrated that HRES-1/Rab4 regulates the expression of CD4 by inhibiting its surface expression on peripheral blood CD4^+^ T cells and targeting it for lysosomal degradation, which may be one of the mechanisms through which HRES-1/Rab4 inhibits HIV infection (49). In addition, the expression of HERV-K (HML-2) Env was significantly increased in neuronal cells from PLWHs (50). Neurons expressing high levels of HERV-K (HML-2) Env exhibited elevated levels of nerve growth factor and brain-derived neurotrophic factor (50). Further studies revealed that HERV-K (HML-2) Env enhances the survival rate of neuronal cells exposed to HIV Vpr (50). Although the widespread global spread of HIV and the incidence of HIV/AIDS-associated brain lesions suggest limitations to this inhibitory effect, the ability to enhance this effect through protein engineering may provide new ideas for the field of HIV therapy. Here, based on the above discussion and possible subsequent references, we have constructed Fig. 2 to illustrate relevant findings and potential research directions.

## IMPACT OF HERVs IN HIV-PIR

Some PLWHs may experience PIR after initiating ART, despite successful viral suppression. The mechanisms underlying PIR are attributed to reduced bone marrow hematopoietic function, insufficient thymic output, apoptosis, senescence, abnormal immune activation, cytokine dysregulation, and the persistence of viral reservoir (51). Damage to central immune organs and a dysbalanced immune response are considered primary causes of PIR. As mentioned earlier, HERVs can also be detected in PLWHs undergoing successful ART, but their expression levels are typically lower compared to those in PLWHs who have not received ART or in those with treatment failure (13–15). HERVs are believed to interfere with various human biological processes through the expression of retroviral genes, rearrangement of genomic loci following reverse transcription, or regulation of neighboring gene transcription via their LTRs (52). HERVs LTRs contain promoter and enhancer elements that influence the expression of adjacent genes (52, 53). It has been reported that HERV LTRs contribute to approximately 15–30% of immune cell enhancers, particularly near genes related to immune response and cellular stress (54, 55). Therefore, we hypothesize that HERVs may be involved in the development of HIV-related PIR, primarily through their role in mediating abnormal immune activation.

### HERV-mediated adaptive immune responses in PLWHs

It has been reported that specific CD8^+^ T cell responses against HERVs (HERV-K/L/H/W) peptides can be detected in PLWHs (56). Subsequently, the researchers measured the HERV-L IQ10-specific T cell responses in three longitudinally observed PLWH participants. The results demonstrated a sustained, independent, and high-intensity HERV-L IQ10-specific T cell response, which showed a decline following the initiation of ART (56). Further studies revealed that the proportion of terminally differentiated cells was higher among HERV-L IQ10-specific CD8^+^ T cells, which were able to kill B cell targets presenting homologous peptides (56). In a subsequent study, the researchers measured the HERVs (HERV-K/L/H) peptide-specific CD8^+^ T cell responses in elite controllers, virologic noncontroller, immunologic progressors, and ART-suppressed individuals (57). The results showed that elite controllers exhibited significantly higher HERVs peptide-specific CD8^+^ T cell responses compared to the other groups (57). Additionally, the study found that HERVs peptide-specific CD8^+^ T cells displayed a higher degree of differentiation and a lower activation level but were capable of producing dual cytokines (IFN-γ and TNF) (57). Furthermore, a study on the T cell responses to HERV peptides in children with mother-to-child HIV transmission showed that out of 42 participants, 26 exhibited HERVs (HERV-K/L/H) peptide-specific CD8^+^ T cell responses, with the strongest response observed against HERV-L (58). These studies demonstrate that HERVs peptide-specific CD8^+^ T cell responses are negatively correlated with HIV viral load and positively correlated with CD4^+^ T cell counts (56–58). Subsequent studies isolated HERV-K (HML-2)-specific CD8^+^ T cells from PLWHs and found that these cells exhibited Vif-dependent responses to HIV-infected cells *in vitro* (59). However, other studies have indicated that the detection rate of HERV-K (HML-2)-specific T cell responses is relatively low in PLWHs (60). Therefore, current research suggests that members of the HERV-K, -L, -H, and -W families elicit specific T cell responses in PLWHs, which are closely linked to the immune status of the individual. Moreover, the intensity of HERV-specific T cell responses is influenced not only by viral load and immune status but also by individual variability, possibly resulting from incomplete immune tolerance to HERVs (61).

Likewise, studies have found the presence of HERV-specific antibodies in PLWHs, although the antibody activity varies depending on the different peptide regions and epitopes, with antibody titers gradually decreasing during HIV infection (62–68). Compared to healthy individuals, PLWHs exhibited a significantly reduced response to HERV-K Gag peptide 137, while the response to peptide 157 was significantly elevated; in elite controllers, a significant reduction in response to peptide 85 was observed, along with a significant increase in response to peptides 81 and 117 (68). Besides, the study found a significant correlation between anti-HERV-K Gag antibodies and T cell responses (68). Researchers have detected the specific antibodies against the transmembrane (TM) region of the HERV-K Env protein in PLWHs, which have been observed to enhance the cytotoxic activity of natural killer (NK) cells against infected cells (66). Michaud et al. reported a significant increase in antibody titers against the TM region of the HERV-K Env protein in PLWHs compared to uninfected adults, with the highest titers observed in “elite controllers” (67). Conversely, another study found that patients in the advanced stages of HIV disease progression display elevated antibody titers associated with HERVs (64). Thus, high-titer HERV-specific antibodies may act as a double-edged sword, potentially aiding in the clearance of HIV-infected cells while also contributing to abnormal immune-mediated cytotoxicity.

The findings suggest that, in the context of HIV infection, HERVs can trigger an adaptive immune response, potentially aiding in the clearance of HIV-infected cells. As such, screening for antigenic peptides, antibodies, and T cells of HERVs may open new avenues for HIV immunotherapy. Nevertheless, the efficacy and potential risks of the adaptive immune response induced by HERVs are not yet fully understood. An excessive immune response could be detrimental to the immune reconstitution of PLWHs.

### HERV-mediated inflammatory responses in PLWHs

Similar to exogenous retroviruses, HERVs' dsRNA and cDNAs can activate the interferon-stimulated genes (ISGs) pathway and induce a type I interferon (IFN- I) response (9). Equally, as shown by Fang et al., the activation of HERVs induces the release of interferons and activates CD4^+^ T/CD8^+^ T cells, playing an important role in antitumor immunity (69). A ChIP-Seq analysis of three human cell lines treated with IFN-γ revealed that 27 transposable element families were enriched in IFN-γ-induced binding peaks, with 20 of these originating from HERV LTR promoter regions (54). Further gene ontology (GO) term analysis revealed that HERV LTR-associated sequences are enriched alongside genes involved in cytokine and inflammatory responses, which may participate in innate immunity, particularly the IFN-γ response (70). Additionally, HIV infection of primary CD4^+^ T cells triggers activation of HERV9 LTR-containing transcriptional start sites (71). Further analysis revealed that HIV-induced HERVs with TSSs were predominantly enriched near immune-related genes (71). Meanwhile, in HIV-infected primary CD4^+^ T cells, HIV indirectly activates LTR12C, driving the transcription of downstream antiviral genes GBP2 and GBP5 (71). Moreover, during HIV infection, upregulation of DHRS2 and SEMA4D genes fused with upstream LTR12D and LTR12C sequences may contribute to the increased immune response exhaustion and aging observed post-infection (71). In studies on the integrity of T helper (Th) cell lineage differentiation in mice, the histone methyltransferase SETDB1 was found to suppress the expression of ERV LTRs, which function as enhancers for Th1 cell polarization genes, through the deposition of H3K9me3 marks, thereby shaping and regulating the Th1 gene network (72). PLWHs with PIR exhibit higher proportions of T cell exhaustion and senescence, with their CD4^+^ T cells more prone to polarizing into pro-inflammatory Th cells, leading to dysregulated cytokine secretion (73).

Although there is no direct evidence linking HERVs to the formation of PIR, detectable HERV expression in PLWHs receiving ART suggests a potential role in post-treatment immune reconstitution. The positive feedback loop between HERVs and inflammation, along with their influence on T cell polarization, further supports this speculation. Future studies may explore the use of HERVs as biomarkers for HIV-associated inflammation and PIR, offering tools to predict and manage excessive immune responses in clinical settings.

## HERVs AND HIV RESERVOIRs

The eradication of HIV reservoirs represents the primary obstacle to the development of curative therapies. The persistence of these viral reservoirs also contributes to incomplete immune reconstitution. HIV reservoir formation begins in the early stages of infection and continues throughout treatment (3, 74). In response, some researchers have proposed the strategy of achieving complete silencing of integrated HIV. As endogenous retroviruses that have successfully integrated and remained silent in the human genome, HERVs may offer valuable insights into mechanisms for silencing HIV.

Previously, Amabile et al. employed the silencing mechanisms of HERVs to construct an inhibitory complex comprising an effector domain that recruits KRAB-ZFPs, DNMT3A, and DNMT3L (75). This complex was successfully employed to maintain the repression of genes such as B2M, I FNAR1, and VEGFA in somatic cells (75). Additionally, the human silencing hub complex is known to protect the genome from the intrusion of both endogenous and exogenous retroviruses, as well as non-retroviruses elements, by regulating the dissemination of integrated, RNA-derived mobile elements within the host genome (76). These findings suggest that the human genome possesses innate protective mechanisms that regulate gene expression and prevent the invasion of foreign nucleic acids. Further exploration and application of these mechanisms could offer potential strategies for HIV suppression in the future.

## CONCLUSIONS

HIV is a retrovirus that targets the immune system, specifically CD4^+^ T cells, leading to a decline in immune function that can result in the development of AIDS. The global prevalence of HIV/AIDS presents a major public health challenge. Additionally, humans have previously encountered other retroviruses, and the remnants of these retroviruses within the human genome, known as HERVs, form a set of transposable elements. These elements may potentially offer some protection against exogenous retroviruses like HIV. However, the widespread dissemination of HIV suggests that HERVs do not provide a comprehensive defense against HIV infection. Currently, HIV prevention and treatment follow a “test and treat” approach, with antiretroviral drugs targeting different stages of HIV replication, successfully suppressing viral replication within months. As a result, the potential antiviral role of HERVs in the context of HIV infection, particularly their actual impact in physiological environments, remains inadequately defined. Presently, challenges in HIV treatment include drug resistance, PIR, and the persistence of HIV reservoirs. The precise role of HERVs in these processes remains unclear and requires further investigation.

In this GEM, we explore the impact of HIV infection on the expression of HERVs and their potential mechanisms, with a particular focus on the role of HERVs in the formation of PIR and viral reservoirs. However, due to the limited research available, no definitive conclusions can yet be drawn. Thus, a comprehensive understanding of the regulatory networks governing HERVs and the mechanisms underlying their interaction with HIV is essential to fully assess their potential for use in clinical HIV treatments.
